# A protocol for evaluating a multi-level implementation theory to scale-up obstetric triage in referral hospitals in Ghana

**DOI:** 10.1186/s13012-020-00992-2

**Published:** 2020-05-12

**Authors:** Caitlin R. Williams, Stephanie Bogdewic, Medge D. Owen, Emmanuel K. Srofenyoh, Rohit Ramaswamy

**Affiliations:** 1 Department of Maternal and Child Health, Gillings School of Global Public Health, University of North Carolina at Chapel Hill, Chapel Hill, North Carolina USA; 2 Wake Forest School of Medicine, Winston-Salem, North Carolina USA; 3 Kybele, Inc., Lewisville, North Carolina USA; 4 Greater Accra Regional Hospital, Accra, Ghana; 5 Public Health Leadership and Maternal and Child Health, UNC/RTI Consortium for Implementation Science, Gillings School of Global Public Health, University of North Carolina at Chapel Hill, Chapel Hill, North Carolina USA

**Keywords:** Ghana, Low- and middle-income countries, Obstetric triage, Scale-up, theory of change, Maternal Newborn Health, implementation theory, evaluation, frameworks

## Abstract

**Background:**

Ghana significantly reduced maternal and newborn mortality between 1990 and 2015, largely through efforts focused on improving access to care. Yet achieving further progress requires improving the quality and timeliness of care. Beginning in 2013, Ghana Health Service and Kybele, a US-based non-governmental organization, developed an innovative obstetric triage system to help midwives assess, diagnosis, and determine appropriate care plans more quickly and accurately. In 2019, efforts began to scale this successful intervention into six additional hospitals. This protocol describes the theory-based implementation approach guiding scale-up and presents the proposed mixed-methods evaluation plan.

**Methods:**

An implementation theory was developed to describe how complementary implementation strategies would be bundled into a multi-level implementation approach. Drawing on the Interactive Systems Framework and Evidenced Based System for Implementation Support, the proposed implementation approach is designed to help individual facilities develop implementation capacity and also build a learning network across facilities to support the implementation of evidence-based interventions.

A convergent design mixed methods approach will be used to evaluate implementation with relevant data drawn from tailored assessments, routinely collected process and quality monitoring data, textual analysis of relevant documents and WhatsApp group messages, and key informant interviews. Implementation outcomes of interest are acceptability, adoption, and sustainability.

**Discussion:**

The past decade has seen a rapid growth in the development of frameworks, models, and theories of implementation, yet there remains little guidance on how to use these to operationalize implementation practice. This study proposes one method for using implementation theory, paired with other kinds of mid-level and program theory, to guide the replication and evaluation of a clinical intervention in a complex, real-world setting. The results of this study should help to provide evidence of how implementation theory can be used to help close the “know-do” gap.

**Plain language summary:**

Every woman and every newborn deserves a safe and positive birth experience. Yet in many parts of the world, this goal is often more aspiration than reality. In 2006, Kybele, a US-based non-governmental organization, began working with the Ghanaian government to improve the quality of obstetric and newborn care in a large hospital in Greater Accra. One successful program was the development of a triage system that would help midwives rapidly assess pregnant women to determine who needed what kind of care and develop risk-based care plans. The program was then replicated in another large hospital in the Greater Accra region, where a systematic theory to inform triage implementation was developed.

This paper describes the extension of this approach to scale-up the triage program implementation in six additional hospitals. The scale-up is guided by a multi-level theory that extends the facility level theory to include cross-facility learning networks and oversight by the health system. We explain the process of theory development to implement interventions and demonstrate how these require the combination of local contextual knowledge with evidence from the implementation science literature. We also describe our approach for evaluating the theory to assess its effectiveness in achieving key implementation outcomes. This paper provides an example of how to use implementation theories to guide the development and evaluation of complex programs in real-world settings.

Contribution to the field

- There has been a rapid growth in the development of frameworks, models, and theories of implementation. Yet, much is unknown about how these theoretical developments can inform implementation practice.
- By proposing a method for integrating implementation, mid-level, and program theory to guide the development and evaluation of a complex scale-up project, this study protocol responds directly to recent calls to operationalize theory and develop pragmatic, yet rigorous, methods for assessing theory and informing practice.
- The results should provide empiric evidence of how implementation theory can be used to accelerate progress on getting potentially life-saving interventions into the field.

## Background

The Millennium Development Goals (MDGs) sought to decrease global maternal mortality by 75% and under-five mortality by two-thirds between 1990 and 2015 [1, 2]. During this time period, the global maternal mortality ratio decreased by nearly 44% [3]. However, progress was uneven and by 2015, an estimated 66% of maternal deaths occurred in Sub-Saharan Africa [3]. Under-five mortality followed similar trends, showing heterogeneity across regions and countries, despite an overall 53% reduction in the global under-five mortality rate [4]. Progress on reducing newborn mortality and stillbirth was particularly slow [5]. Reducing maternal and newborn mortality, as well as stillbirth, represents a key unfinished part of the MDG agenda that is carrying into the Sustainable Development Goals (SDG) era.

Ghana significantly reduced maternal and newborn mortality during the MDG period (from 634 maternal deaths per 100,000 live births in 1990 to 319 in 2015; from 42 newborn deaths per 1000 live births in 1990 to 28 in 2015) [3, 6]. Efforts focused primarily on increasing facility births with a skilled attendant and referring high-risk cases to higher-level hospitals. Yet data on maternal and newborn outcomes within facilities suggest that the quality and timeliness of care needs to improve in district and regional facilities who serve large volumes of mothers needing specialized care [7, 8]. Reducing the “third delay”—the delay in receiving adequate and appropriate treatment once care is sought [9]—is of particular priority.

The third delay begins when a pregnant woman arrives at the hospital, with an important component being delay in first assessment upon arrival [10]. The potential consequences of this delay can be severe for women with high-risk or otherwise complicated pregnancies and their infants. Therefore, quickly and accurately assessing women upon their arrival to the facility and developing tailored, risk-based care plans constitute an important first step in assuring positive outcomes [11].

Beginning in 2013, the Ghana Health Service (GHS) and Kybele, a US-based non-governmental organization, developed an obstetric triage system for quick and accurate assessment of obstetric patients in low-income settings [11] as part of a long-term partnership to improve maternal and neonatal outcomes [11–13].

The core elements of the system were developed and tested at Greater Accra Regional Hospital (GARH) (formerly Ridge Regional Hospital), a tertiary facility, between 2012 and 2015. The primary focus of the GARH project was to test and refine the clinical aspects of the obstetric triage protocol intervention, with timeliness of assessment as the clinical intervention outcome of interest. Implementation resulted in a reduction in median waiting time from arrival to assessment from 40 to 5 min [11].

In 2018, the obstetric triage system was introduced at Tema General Hospital (Tema), another high-volume facility in the Greater Accra region. The objective of this pilot was to gain knowledge about other clinical and implementation outcomes relevant to triage. In addition to timeliness of assessment, the Tema implementation also measured the accuracy of triage assessment (i.e., were women assigned the correct risk category) as a clinical outcome. Recognizing that neither outcome could be achieved without a systematic implementation process, a theory was developed to guide implementation. Fidelity, adoption, and sustainability were measured as implementation outcomes. The median arrival to assessment time decreased from 1 h and 8 min to 9 min within 4 months, and the overall time needed to achieve full implementation decreased from 3 years in GARH to 5 months in Tema. Data on the other clinical and implementation outcomes have been collected and are being analyzed.

Based on the Tema results in improving timeliness of assessment, in 2019, Kybele and GHS began the Obstetric Triage Implementation Package (OTIP) project to scale the triage system to six additional Ghanaian hospitals. Implementing evidence-based practices at scale requires simultaneous attention to systems change across multiple levels [14]. Thus, the “within-facility” theory of change developed at Tema was augmented to include components across facilities and across the health system. The resulting multi-level theory posits how multiple facilities might share learnings and build “across-facility” synergy, thus producing higher-fidelity implementation and clinical quality more quickly than a single facility attempting to embed the practice on its own. This augmented multi-level implementation theory (described in detail and diagrammed in Fig. 2 below) will be used as a process model to guide implementation and will also serve as the evaluation framework for the implementation process. The objective of this evaluation is twofold: to assess the effect of this theory on implementation fidelity and triage quality, and to use this assessment to generate evidence for a generalizable mid-level theory for implementing evidence-based interventions in low-resource maternity settings.

## Methods

### Design

The OTIP evaluation uses a type II hybrid effectiveness-implementation pre-post design [15] focusing on both clinical and implementation outcomes. Implementation is staggered across facilities, starting two at a time every 6 months. This staggered approach allows for before-after comparisons of facilities with their own results, as well as cross-facility comparisons of the implementation process. The first two facilities began triage implementation in late August 2019. In the initial 6 months of implementation, facilities receive intensive implementation support, with facilities then transitioning into full self-management of the program.

The evaluation questions of interest are as follows:

1. To what extent were the components of the implementation theory used in guiding the implementation process?
2. How effective was this theory in accelerating and sustaining the implementation of obstetric triage? What were the mechanisms by which this occurred?
3. To what extent did the theory result in changes beyond the level of individual facilities (e.g., changing norms or policy across multiple facilities)?
4. What insights garnered from this specific implementation theory can be generalized to other settings, helping to define a mid-range theory about the implementation of evidence-based interventions in new settings?

### Setting

As described above, the obstetric triage system was developed at GARH, and replicated at Tema, both high volume obstetric referral hospitals in the Greater Accra region. The six scale-up facilities included in this implementation study are public hospitals that similarly receive large numbers of obstetrics patients (each hospital conducts 4000–7000 births per year). Participating facilities were selected by a national Technical Advisory Group (TAG) consisting of leaders from the GHS responsible for maternal health and institutional care (the structure and role of the TAG is discussed below). Priority was given to high-volume facilities with Comprehensive Emergency Obstetric Care capability.

### Intervention

**Fig. 1:**
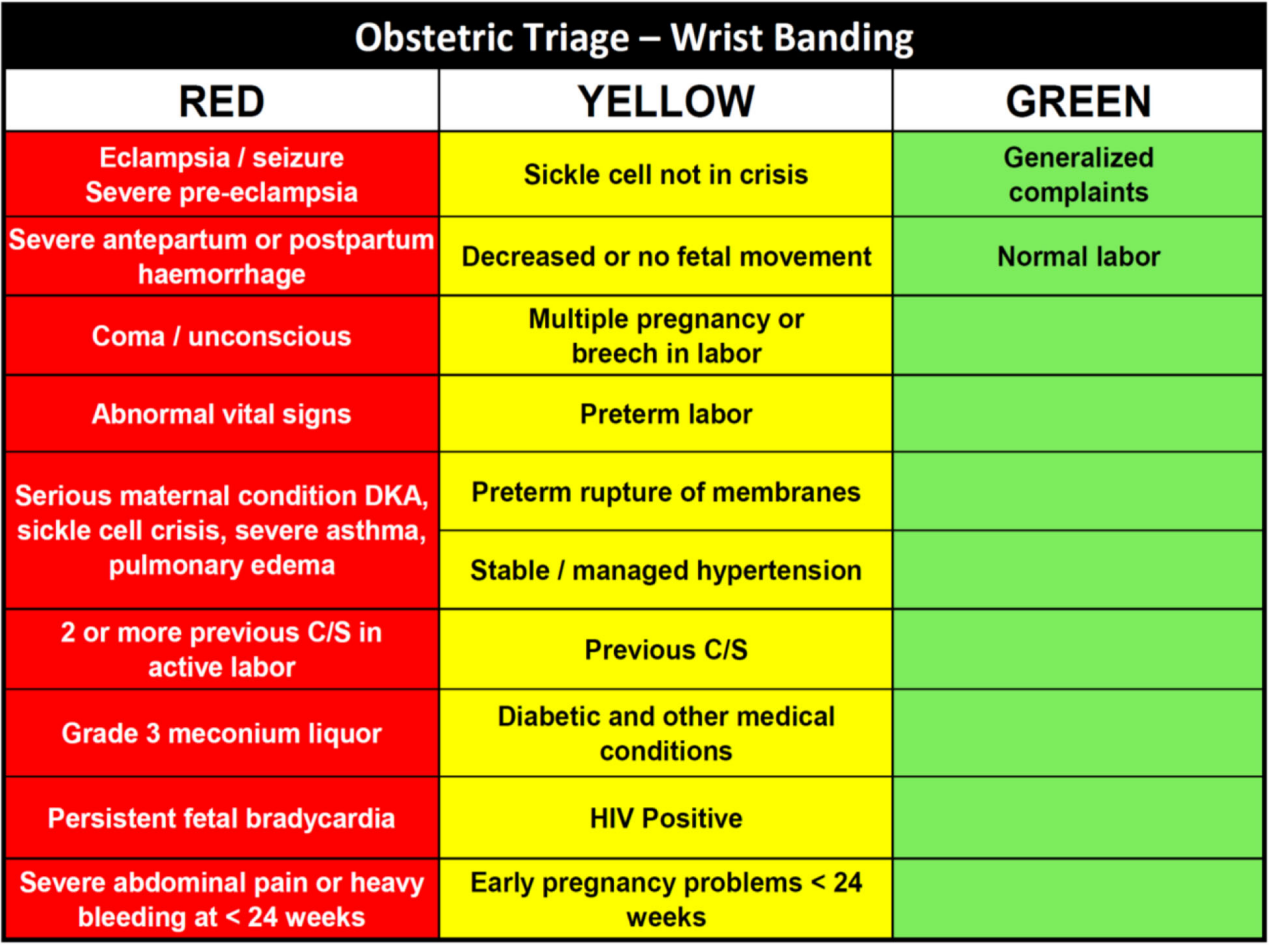
OTIP risk classification for banding and care-plan development

The obstetric triage system is a midwife-led clinical assessment and prioritization intervention. When an obstetric patient arrives at the facility, she is directed to a triage area for assessment by a midwife. The midwife records the patient’s obstetric and medical history, vital signs, and labor progress onto a standardized triage assessment sheet, leading to categorization of high (red), intermediate (yellow), or low (green) risk, and the application of a corresponding color-coded patient wristband. Based on the diagnosis and risk status, a care plan is developed and documented. High-risk pregnancies require immediate intervention, intermediate-risk cases require careful and frequent monitoring, and low-risk cases proceed with normal childbirth, with the assistance of a midwife. Figure 1 shows the risk categorization for common conditions.

Despite the simplicity of this intervention, it is not commonly used in Ghanaian hospitals, and there are no dedicated triage areas. Assessment typically occurs once a bed becomes available on a first-come, first-serve basis, resulting in delays that can endanger mothers’ and newborns’ lives. Thus, the triage system was developed to facilitate rapid and accurate patient assessment and care planning as a routine part of midwifery practice. Similarly, the implementation theory was developed to facilitate accurate and consistent implementation of the triage system, to help embed triage and care-plan development as a normal and integral part of midwives’ way of work.

### Implementation

The focus on theory in implementation science has grown rapidly over the past decade but has primarily remained in the domain of research [16]. The systematic use of mid-level and program theories has been proposed as one way to bridge implementation theory and practice, but practical guidance to practitioners on how to create or select theories has been limited [17]. In a recent commentary, Kislov and colleagues encourage theory building in implementation science to shift from a top-down, static process, to an empirical, iterative process refined by learning from the local environment [17]. Our approach to theory building has adopted this concept, by using a program theory of change as the starting point for a more general implementation theory.

#### Comprehensive implementation theory

As mentioned previously, the implementation theory for OTIP was developed using a theory of change process [18]. Developing a theory of change to guide program design is a key first step in public health planning and evaluation, but this language is not common in the implementation science literature [19]. A key benefit of theory of change methodology is that it requires program designers to specify each step of the change process, and why proposed actions should be expected to produce desired results. Mapping a theory of change helps illuminate important assumptions and hypotheses.

Historically, theory of change methodology has described why an intervention should be expected to produce a desired programmatic outcome [18]. We applied this methodology in partnership with frontline staff in Tema to explore implementation strategies that could be expected to produce the desired implementation outcomes of adoption and fidelity. This resulted in the development of a within-facility implementation theory. In developing the OTIP scale-up theory, the original Tema within-facility theory was validated and supplemented with additional cross-facility and system components based on references to the implementation science literature. We followed this inductive/deductive approach because we needed to develop a facility level implementation process that would generate the engagement and support of staff, and would allow us to incorporate local contextual factors hypothesized to affect implementation.

**Fig. 2:**
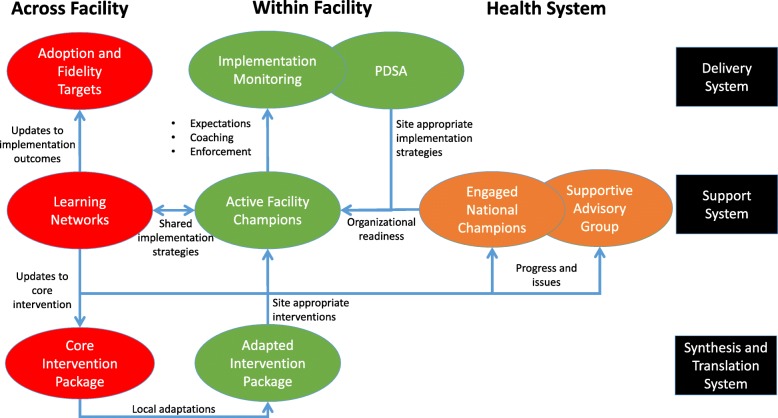
OTIP multi-level implementation theory

The multi-level implementation theory developed to guide OTIP is depicted in Fig. 2. The Interactive Systems Framework (ISF) provides useful language for thinking through how each set of actors contributes to the implementation of OTIP and, ultimately, embedding the new way of work within hospitals [20]. The ISF defines three types of systems in any implementation effort (the synthesis and translation system, the support system, and the delivery system) that work in concert to promote the adoption and embedding of a new intervention or practice. Within OTIP, synthesis and translation system activities include the codification and standardization of the intervention package and its adaptation to local facility contexts (with subsequent modifications to the core intervention package made, as needed, based on implementation experience in each additional facility). Support system activities include selecting participating facilities, strengthening organizational readiness, and building implementation capacity. Delivery system activities include fidelity monitoring, coaching and mentoring of staff, and the adoption of strategies to improve fidelity and quality of implementation.

#### ***“Withi***n-facility” theory

The within-facility implementation theory has its roots in the Evidence Based System for Implementation Support (EBSIS) described by Wandersman and colleagues, which emphasizes the need for training, technical assistance (TA) tools, and quality improvement (QI) to support the implementation of an evidence-based intervention [21]. In addition, it relies on the findings of Aarons et al. that leaders within organizations and systems play a large role in defining implementation climate and readiness [22, 23].

Application of the theory begins with the selection of triage champions. These are midwives and physicians selected by the leadership in each facility to act as facilitators, change agents, and implementation coaches. The expectation is for them to take on what Aarons et al. describe as a transformational leadership role [23]. As OTIP is a midwife-led intervention, the midwife champions become experts on the obstetric triage system and provide formal training for their peers. Midwife champions also provide crucial peer leadership to ensure acceptance and adoption of the intervention in each facility through fidelity monitoring. The physician champions are responsible for garnering the buy-in of other physicians in their hospitals. Each participating facility differs in staff composition and daily operations. Thus, the OTIP implementation theory proposes that while all facilities should receive identical training and tools, equipping champions to develop their own approach to TA and QI is more likely to be successful than mandating a one-size-fits-all implementation approach.

**Fig. 3:**
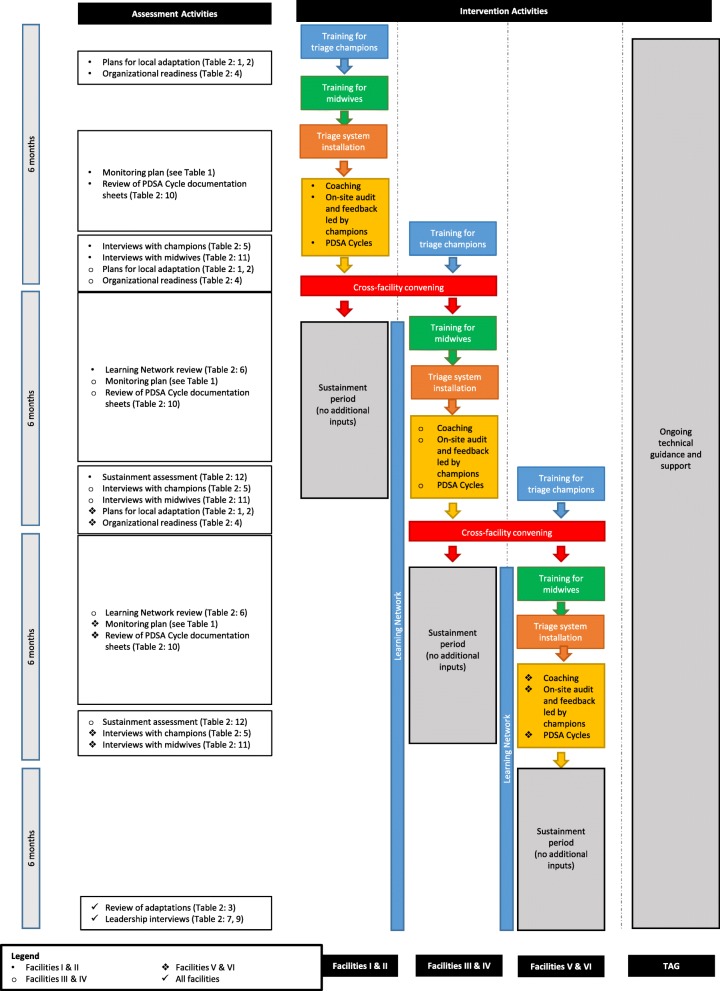
Overview of OTIP activities

Once selected, triage champions undergo intervention-specific training for 2 days, delivered initially by Kybele and subsequently by national champions, whose role is described below. Training on the first day focuses on the clinical aspects of triage, delivered through a combination of didactic and hands-on formats. Training on the second day covers performance targets, fidelity monitoring, and adaptation. Champions are trained on a phased monitoring approach (Fig. 3) to measure compliance against three intervention quality standards related to clinical outcomes: banding compliance (was every mother banded?), banding accuracy (was the correct band assigned based on the assessment?), and the creation of the care plan (was the mother’s care plan aligned with the band and diagnosis?). The standards are shown in Table 1. Training is also provided during day two on the use of Plan-Do-Study-Act (PDSA) cycles [24] to hypothesize causes for gaps in intervention quality, and to develop and test locally appropriate strategies to address them. A PDSA Cycle worksheet is provided for documentation of each cycle, learning, and subsequent adaptations of implementation strategies to improve quality adoption of triage.

**Table 1:** Monitoring standards for each phase

Phase	Metric	Target (Each target should be achieved for 3 consecutive weeks before beginning the next phase of monitoring)
Phase 1, banding compliance	% of patients banded (# of patients banded per shift/# of patients admitted per shift)	Target depends on facility size (births per year): 2000–3000, 90%; 3000–5000, 85%; > 5000, 80%
Phase 2, triage assessment form compliance	% of reviewed forms with relevant fields completed and risk-based care plan entered (from a random selection of 10 triage assessment forms each week)	100%
Phase 3, banding accuracy	% of reviewed forms with correct color-code of band based on triage assessment and OTIP risk-assessment/banding classification (from a random selection of 10 triage assessment forms each week)	90%

After training, the champions determine the best method for delivering the training content to their colleagues in each hospital, as well as where and how to prepare the triage area. As described by Hawe and colleagues, these adaptations target the *form* of the intervention, while retaining its *function* [25]. In other words, the core intervention activities (assessment, completion of triage form, wrist-banding, care plan development) are identical, but the manner in which the triage space is designed and situated is determined locally. Similarly, the function of the implementation strategies is standardized across all participating facilities (e.g., training on triage system, coaching to enforce proper practice), but the form is permitted to vary according to champions’ understanding of what will work best in their facilities (e.g., classroom sessions vs. on-the-job training, individual coaching at the bedside vs. role play sessions in team meetings).

Following the kick-off Kybele-led training, facility champions are provided access to the implementation monitoring system (developed using a Google application) through their smart phones. Each week, a sample of OTIP triage assessment forms is randomly selected from the previous 7-day period. Depending on the monitoring phase, champions enter data on banding compliance, triage assessment form completion, and banding accuracy into the system. For each facility, the system aggregates the data to assess weekly compliance against the standard, creating run charts of performance over time. These charts are visible to all facilities and to the evaluation team. Each week, the facility champions review the data and use PDSA cycles to design new implementation strategies, or to reinforce previous ones to improve implementation performance against the standards. These strategies primarily fall within the categories described by Leeman et al. as dissemination strategies (messages, communication, posters, recognition, enforcement) to improve the knowledge and attitude of frontline staff and capacity building strategies (additional training, job aids, coaching) to improve their capability to perform triage [20]. One standard is addressed in each monitoring phase, and the monitoring proceeds to the next phase when compliance to the standard is achieved for three continuous weeks. Once phase 3 standards have been met for three continuous weeks, the facility is categorized as having achieved full implementation. When the implementation in the facility is considered complete, a close out event is conducted in each facility, in which each facility develops an ongoing monitoring plan. No additional support is provided for the implementation of this plan.

#### “Across-facility” theory

In their 2004 systematic review of the diffusion literature, Greenhalgh and colleagues highlighted the importance of social networks, opinion leaders, and champions to facilitate the spread of innovations [26]. The across-facility theory operationalizes these ideas around social networks and dissemination through the use of a champion-led learning network. The network is housed on a WhatsApp platform and provides triage champions the opportunity to share learning and insights, ask for guidance, collectively problem-solve, and create healthy peer competition to sustain implementation. In addition, the clinical champions convene in-person when each new set of facilities comes on board to share successful practice. The primary objective of these learning sessions is to document successful implementation strategies, to create a formal implementation guide that will inform future scale-up, and to discuss adaptations to enhance implementation outcomes such as adoption, penetration, and sustainability.

#### System-level theory

Implementation theory at the system level involves dissemination and communication of implementation progress to national-level stakeholders to encourage adoption of the innovation nationwide. For OTIP, this process involves the establishment of a Technical Advisory Group (TAG), consisting of leaders from GHS’s Institutional Care Division as well as others responsible for developing policies, practices, and programs to improve clinical care delivery nationwide. To promote collaboration across levels, one midwife champion and one physician champion are designated as “national champions” and TAG members. The TAG also includes selected facility leaders (e.g., head of obstetrics) to further reinforce the relationship between the national and facility levels. The TAG provides transactional leadership [23] through monitoring and supervision of overall implementation. The TAG meets regularly throughout the project period to discuss implementation challenges, share lessons learned, and build a collective understanding of how to successfully integrate the obstetric triage system into national policy.

As described by Raghavan and colleagues [14], policies need to be deployed across multiple levels of the policy ecology. As insights about requirements for successful implementation are shared with the TAG, policy advocacy may need to encompass, for example, hiring or reassigning staff across maternity units for triage, issuing standards for equipping dedicated triage areas, encouraging facility leaders to promote triage within their institutions, and establishing national standards for selecting triage champions. We use the word “policy” broadly to include both formal policy change as well as changes in rules, customs, or traditions that arise due to informal influence exerted by the TAG, even if formal institutional policies are not developed or rewritten.

### Evaluation approach

A convergent design mixed methods approach will be used [27], as is common in the evaluation of complex multi-level program implementation. Relevant data will be drawn from tailored assessments, routinely collected process and quality monitoring data, textual analysis of relevant documents and WhatsApp group messages, and key informant interviews. Evaluation at the facility level will include process evaluation during the first 6 months of implementation in each facility, and outcome and sustainability evaluation after external support for implementation has ended. Cross-facility comparisons and assessment of the TAG support will be undertaken at the end of the project period, after external support for implementation has concluded in all facilities

#### Within-facility assessments

The primary clinical outcome is the waiting time from patent arrival to triage assessment. At each facility, waiting times are collected before, after, and 6 months following the end of receiving intensive triage implementation support from Kybele, to assess sustainment following the cessation of external implementation support. If there is no triage program in the facility at baseline, data from arrival to when the patient is first seen by a midwife is used to calculate waiting time. Median waiting times are compared in each facility. A priori sample size calculations were conducted using G*Power. Assuming an effect size similar to the pilot study (*d* ≈ 0.776), a minimum of 60 women will need to be included in each round of data collection in each facility, in order to detect a change with 95% power. Given the potential for a smaller effect size than observed in the pilot, 75 women will be recruited in each round of data collection. The secondary clinical outcomes are assessment accuracy and appropriateness of the care plan developed given the diagnosis. As data collection on these secondary outcomes cannot occur until the triage program is implemented, changes cannot be compared to baseline, but data on these outcomes will be collected at endline and 6 months following the end of receiving implementation support.

The primary implementation outcomes are *acceptability*, *adoption*, and *sustainability*. Acceptability and adoption of the obstetric triage system by frontline staff at each facility will be measured through semi-structured interviews based on the Consolidated Framework for Implementation Research (CFIR) constructs, and by measures of banding compliance at endline. Interviews will be conducted by a senior member of the research team, and their notes will be coded by a team of graduate-trained coders using a codebook generated inductively based on interview data from the first two implementing facilities. These codes will be used to categorize the interview data from the remaining facilities, and additional inductive codes will be added. Changes in banding compliance over time will be assessed using run charts. Sustainability will be assessed using Normalization Process Theory (NPT), which seeks to explain how new technologies and practices become embedded into health provider routines [28, 29], as well as by the post-implementation measurements of waiting time, compliance, and accuracy. A recent systematic review found that NPT assessment can provide critical information to determine the extent to which an implementation project will be successful from a sustainability perspective [30]. While NPT assessments have primarily been conducted through qualitative data collection, NPT developers assert that the theory holds a degree of flexibility, and data collection can be adapted to best meet contextual needs [30]. Additionally, NPT developers have introduced the NoMad quantitative instrument intended assess intervention integration [31]. The NoMad tool has been used in global health to assess the degree to which a program has been embedded within a given context during implementation [32]. The NoMad will be adapted, per the developers’ instructions, to ensure it is relevant to the project and context-specific. Approximately 6 months after the end of receiving implementation support from Kybele, all frontline staff will complete an NPT assessment to measure the four key components of NPT, which include coherence, cognitive participation, collective action, and reflexive monitoring [33].

Implementation determinants posited to affect the outcomes will also be measured. The CFIR-based interviews will also be used to measure staff perceptions of barriers to implementation. In addition, following training of the triage champions, but prior to implementation, a facility readiness assessment will be conducted using a modified version of the *R* = MC^2^ readiness heuristic [34, 35]. This version is the result of a Delphi process with content experts to validate and simplify the items within *R* = MC^2^ [35] and make it more pragmatic [36]. This assessment has been further collaboratively adapted with the triage champions for use in Ghana. As readiness is posited to affect both the ability of organizations to implement new practices and the degree to which those new practices can be embedded within ways of work, we will explore whether baseline readiness as measured by *R* = MC^2^ is correlated with adoption and sustainability. Given the lack of guidelines around how to interpret *R* = MC^2^ scoring, this part of the study will be exploratory in nature and presented via narrative description rather than statistical analysis. Specifically, we will identify any barriers to readiness captured through the assessment and explore whether these barriers affected the implementation outcomes in any discernable way.

Local implementation strategies developed and adapted through the PDSA cycles will be coded using the FRAME framework [37]. This coding will allow us to assess the nature and types of adaptations undertaken by each facility team. Finally, in-depth interviews will be conducted with the national and facility champions to learn more about their growth as leaders. These qualitative data will be analyzed thematically to evaluate how champions perceive the OTIP as strengthening their leadership capacity, an important factor in implementation readiness and program sustainability.

Assessment of the role of the TAG will be based on a review of meeting minutes and correspondence to describe TAG activities and focus areas. In-depth interviews will be conducted with TAG members at project conclusion to gain insight into their perceptions of the role of the TAG, its success in achieving its aims, and the lessons learned throughout the obstetric triage scale-up project. TAG members will also be asked to reflect on the feasibility, acceptability, and perceived utility of the TAG for further scale-up of the obstetric triage system, as well as other public health projects that GHS might seek to implement. These interviews will be coded inductively and analyzed using thematic analysis.

Table 2 summarizes the data that will be collected at each stage of implementation mapped to the theory shown in Fig. 2. Figure 3 provides an overview of the intervention and assessment activities and illustrates how the data presented in Table 2 will be used for evaluation across the course of the project period.

**Table 2:** Types of data to be collected during OTIP implementation

No.	Implementation system	Level	Data	Frequency	How collected
1	Synthesis and translation	Within facility	Local adaptation of triage site setup	Once in each facility prior to implementation	Visual observation of the layout of each facility and of the triage site
2	Synthesis and translation	Within facility	Local adaptation of training delivery	Once in each facility once champion-led training is complete	Short questionnaire completed by facility champions
3	Synthesis and translation	Across facility	Adaptations to training materials, forms, bands, and other intervention components	Once at the end of the project period	Document review of training materials across training sessions; interview with trainers
4	Support	Within facility	Organizational readiness for implementing OTIP	Once in each facility, following training but prior to implementation start	Readiness instrument adapted from Scaccia [34, 39]
5	Support	Within facility	Facility champion coaching activities, strategies, and results	Once in each facility when phase 3 performance standards are achieved (see Table 1)	Semi structured interviews of facility champions
6	Support	Across facility	Utilization and effectiveness of learning networks	Ongoing throughout implementation and during face-to-face learning network meetings	Review of WhatsApp messages and documentation of discussion during learning sessions
7	Support	System level	Growth in leadership for implementation	Once at the end of the project period	Semi-structured interviews of national triage champions
8	Support	System level	TAG activities	Ongoing throughout implementation	Review of TAG meeting agendas, email correspondence, and meeting minutes
9	Support	System level	TAG perception of roles and influence	Once at the end of the project period	Semi-structured interview of TAG members
10	Delivery	Within facility	Local implementation strategies to meet performance standards	Ongoing throughout implementation in each facility	Review of PDSA worksheets
11	Delivery	Within facility	Staff perception of the implementation process	Once in each facility when phase 3 performance standards are achieved	Semi structured interview of selected frontline staff using adapted CFIR framework
12	Delivery	Within facility	Sustainment of the OTIP process	Once in each facility approximately 6 months after phase 3 performance standards are achieved	NPT assessment instrument

A rich blend of qualitative and quantitative data will be used to answer the research questions. To assess the extent to which the theory was used to guide implementation, an assessment rubric will provide a detailed synthesis of the data in Table 2. This synthesis will seek to determine how strongly the implementation data reflects the strength and utility of each component of the theory for each facility.

One aspect of the implementation theory is the EBSIS approach of providing training, support and tools to develop local implementation strategies. To assess this, we will analyze the adaptations from the PDSA worksheets in each facility (#10 in Table 2) to assess whether there is evidence of use of a systematic iterative approach to create strategies to improve implementation quality (based on data related to the success of previous implementation efforts), whether strategies were developed on an ad hoc basis, or whether diverse strategies were employed at all. Finally, we will analyze the qualitative interview data with frontline staff (#11 in Table 2) as well as the NPT data (#12 in Table 2) to gain insight into how implementation capability gets embedded into everyday workflow in each facility.

#### Across-facility assessments

At the across-facility level, we will assess both outcomes and the implementation process. To evaluate whether the time to triage a patient post-intervention differs by facility, we will pool data on post-implementation clinical outcomes (i.e., wait time to assessment, assessment accuracy, appropriateness of care plan given diagnosis) and estimate a fixed-effects model with facility-specific indicator variables and facility-specific covariates as independent variables. If differences in triage time are seen across facilities, we will triangulate the results with the facility-specific process evaluation data to explore hypotheses about facility effects, and conduct additional explanatory interviews with facility staff as needed.

One hypothesis we will test is whether the use of the theory to guide implementation results in progressive maturity of the implementation process. If this is true, we would expect progress towards outcomes to occur faster in facilities where implementation takes place later in the course of the project, moderated by factors such as readiness. To assess this, we will compare the slopes of median wait time for triage collected at baseline, endline, and 6 months after the conclusion of external support for implementation, controlling for initial readiness (#4 in Table 2). We will use a piecewise linear random effects growth curve model for this analysis, with the independent variable being the timing of implementation start in each facility [38]. We will also evaluate strategies that were common across facilities and investigate whether these were the result of sharing in the WhatsApp platform or at the learning network session (#6 in Table 2), or whether different facilities developed similar strategies independently. This will provide insights into how implementation strategy development can be accelerated by sharing across facilities, and help guide the design of future support systems.

These insights are useful for evaluating the generalizability of this theory to the implementation of other evidence-based interventions, and system effects beyond the facility level. The facility level interviews will be augmented by observations of the TAG process (#8), TAG member interviews (#7), and national champion interviews (#9) to assess the influence of the implementation theory on leadership and higher-level system change.

## Discussion

This protocol describes a complex multi-level implementation theory to derive testable hypotheses around how a focused implementation approach can accelerate adoption and embedding of a new evidence-based intervention. In this way, it offers a model for using program theory to develop testable hypotheses and empiric evidence that can then be used to refine mid-level theory, driving both implementation research and practice forward.

This study faces some limitations with respect to research rigor—the most important being the lack of control groups. This limits our ability to assess the effectiveness of the implementation approach employed on clinical outcomes. However, by developing a theory-based approach for how a complex intervention should be scaled in the field rather than proceeding with an ad hoc approach, this evaluation will contribute useful and novel information about how theory-driven implementation can support actors working to install and embed evidence-based practices in low- and middle-income country settings. Looking beyond the research community, empiric data on the merging of implementation theory and practice can be of great use to policymakers, practitioners, and evaluators, who must contend with the vagaries of real-world implementation contexts.

In short, the results of this study should help to provide empiric evidence of how implementation theory can be used to help close the “know-do” gap, and accelerate progress on getting potentially life-saving interventions into the field.

## Data Availability

The datasets collected and analyzed during the current study will be available from the corresponding author on reasonable request.
